# Central laboratory and point-of-care cardiac marker testing capacity of tertiary hospitals in Nigeria – a multicenter study

**DOI:** 10.4314/ahs.v22i2.28

**Published:** 2022-06

**Authors:** Ijeoma Angela Meka, Ochuko Otokunefor, Asuquo Ene, Olugbenga Olalekan Ojo, Mohammed Manu, Emmanuel Chidiebere Okwara, Daniel Oshi, Martin Chukwuka Ugonabo, Isah Adagiri Yahaya

**Affiliations:** 1 University of Nigeria Faculty of Medical Sciences, Chemical Pathology; 2 University of Port Harcourt, Chemical Pathology; 3 University of Calabar Teaching Hospital, Chemical Pathology; 4 Obafemi Awolowo University Teaching Hospital Complex, Surgery; 5 Imo State University Owerri, Faculty of Basic Clinical Sciences, Chemical Pathology; 6 Ahmadu Bello University Teaching Hospital, Chemical Pathology; 7 The University of the West Indies, Community Health and Psychiatry; 8 University of Nigeria, Department of Chemical Pathology; 9 Aminu Kano Teaching Hospital, Chemical Pathology

**Keywords:** Cardiac marker, tertiary hospital, testing capacity, Nigeria

## Abstract

**Background:**

Cardiovascular diseases are major contributors to morbidity and mortality. It is generally recognized that cardiac markers are of particular benefit in the evaluation of patients with suspected Acute Coronary Syndrome (ACS). Tertiary hospitals, mainly teaching hospitals, are expected to be optimally equipped to offer these services. The study therefore aimed at determining the central laboratory and point-of-care cardiac marker testing capacity of tertiary hospitals in Nigeria.

**Method:**

A cross-sectional survey was carried out in government-owned tertiary hospitals in Nigeria. Data were collected using semi-structured self-administered questionnaires, and analyzed using Stata version 13 (Stata Corp., USA).

**Results:**

A total of 34 hospitals participated in the study. The mean (SD) age of respondents was 43.68 (5.2) years. A total of 19 (55.88%) hospitals were found to have a functional cardiac marker testing facility, either in the form of point-of-care, central laboratory testing or both. Of those without a facility, lack of funds to procure equipment was the major reason given. In hospitals with a testing facility, most testing devices were located in the Central laboratory.

**Conclusion:**

Cardiac marker testing capacity of tertiary hospitals in Nigeria, both in the form of point-of-care and central laboratory testing, was found to be barely adequate. Improvement is needed in this area for better diagnosis and evaluation of patients who need the tests.

## Introduction

A cardiac marker is a laboratory test/investigation useful in detecting Acute Myocardial Infarction (AMI) or minor myocardial injury.^1^ They are proteins released into the blood stream by necrotic myocardium and leaky cell myocyte membranes.^2^ These markers are indeed essential in the timely diagnosis and clinical management of acute coronary syndromes. Several cardiac biomarkers which have been in use include Creatine Kinase (CK-MB), Myoglobin, Troponins and Lactate Dehydrogenase (LDH). Each of the listed cardiac markers has its advantages and shortcomings, however troponin is currently the most highly recommended for diagnosis of acute coronary syndromes.

The inclusion of troponins in the diagnosis of MI is central to the third universal definition of MI propounded by the Joint ESC/ACC/AHA/WHF Task Force. This definition stipulates ‘Detection of a rise and/or fall of cardiac biomarker values [preferably cardiac troponin (cTn)] with at least one value above the 99^th^ percentile upper reference limit (URL)’ amongst other criteria.^3^

The cardiac troponins are the regulatory proteins contained in the contractile proteins of the myocardium. The cardiac troponin (cTn) assays have been one of successful diagnostic investigations which have been developed till date. Troponin has three (3) subunits; Troponin I, Troponin T and Troponin C. Cardiac troponin T (cTnT) and troponin I (cTnI) are cardiac regulatory proteins that control the calcium mediated interaction between actin and myosin, while cTnC is a highly conserved Ca2+ binding subunit.^4^ The measurement of serum cTnI and cTnT has been noted to be superior in terms of sensitivity and secificity to cardiac muscle enzyme measurements in the identification of cardiac muscle damage^5^ hence the preference of Cardiac troponins in the diagnostic criteria for MI. Jishi et al^6^ consequently pointed out in their study that one benefit of use of cardiac troponins over cardiac enzymes was that more patients with chest pain whose di-agnosis of myocardial infarction would have been missed with cardiac enzymes were being diagnosed even in the absence of ST-segment elevation. In line with that, Anderson and Morrow^7^ pointedly noted in their publication that serial measurements of cardiac troponin levels is the preferred biomarker method for differentiating non-STEMI from unstable angina and disorders other than acute coronary syndromes. It is pertinent to note that apart from values above the 99^th^ percentile indicated in the diagnosis of MI, it has been recently documented that low-grade elevations in values of cardiac troponins equally have clinical implications. Welsh et al^8^ in their 2019 study observed that there were distinct causes of low-grade elevations of cardiac troponins. They documented that elevations in cTnI are more strongly associated with some cardiovascular disease (CVD) outcomes, whereas cTnT is more strongly associated with the risk of non-CVD death.

Creatine Kinase-MB (CKMB) is a dimeric enzyme, composed of two subunits (B and M), which catalyzes the reversible phosphorylation of creatine by adenosine triphosphate.^1^ Among the various enzyme cardiac markers, CK appears to be the more sensitive. However, the isoenzyme CKMB offers an improvement in sensitivity and specificity over total CK largely because of its greater concentration in cardiac versus skeletal myocytes. CKMB has a ratio of 5% in skeletal muscle which contributes to its increasing level in trauma and inflammation, hence reducing its specificity. It also has its inability to detect minor myocardial damage, due to its high molecular weight as another limitation. However, Total CK and CK-MB levels are correlated with infarct size and are important predictors of prognosis, and equally valuable in evaluating reperfusion. 9,10 CK-MB subgroup analysis has 91% sensitivity and specificity in the diagnosis of AMI during the first 6 hours, and the determination of the CK-MB relative index (CK-MB/total CK × 100) by measuring CK-MB and total CK is also frequently used for diagnosis of MI. If this index is 2.5% or above, CK-MB is probably of myocardial origin.^11^ Hence Creatine kinase MB (CK-MB) by mass assay, has been recommended as an acceptable alternative when cardiac troponin is not available.^12^ Myoglobin is a cytoplasmic hemoprotein, expressed solely in cardiac myocytes and oxidative skeletal muscle fibers that reversibly binds O2 by its heme residue.^13^ It is one of the best available early markers of AMI within 3 hours after symptom onset. It starts to increase in blood within 2 hours after symptom onset of AMI, peaks at 6–9 hours, and returns to normal within 24 hours. This early release feature of myoglobin is attributed to its small size and localization within the cytosol of the cell.^14^ Its major limitation is its poor specificity due to its presence in skeletal muscle leading to increased serum concentrations in skeletal muscle damage. Severe renal disease also leads to failure of clearance leading to increased serum concentrations. Despite these limitations, in clinical practice, most of the limiting factors can be ruled out by careful history taking, 14 and specificity may equally be increased by inclusion of other diagnostic measures like ECG and more specific cardiac markers like troponins. Ahmad et al^15^ in their publication recommended that because of the initial poor sensitivity of cardiac troponins for AMI, myoglobin should be used in conjunction with cardiac troponin for early detection of AMI.

Lactate Dehydrogenase (LDH) and Aspartate Transaminase (AST) were historically used as cardiac markers but are currently no longer recommended except in very low resource poor environments.

Acute Myocardial Infarction has been cited as one of the major causes of morbidity and mortality worldwide.^16^ In Nigeria, the prevalence of cardiovascular-related deaths has been reported to be on the increase. In a study of out-of-hospital deaths in Lagos, Nigeria, 51.1% were attributed to cardiovascular-related deaths with Myocardial infarction forming 8.9%.^17^

Other studies^18^,^19^,^20^,^21^ conducted in Nigeria have also doc-umented evidences of increasing prevalence of myocardial infarction. Two of such studies 18,19 reported this increasing prevalence in 2005.

It has been documented that because recognition of acute MI is important to prognosis and therapy, measurement of biomarkers of necrosis is indicated in all patients with suspected ACS,^12^ and discharging patients with acute myocardial infarction or unstable angina from the emergency department because of missed diagnoses can have dire consequences.^22^

Therefore, with these observations of increasing prevalence having been documented as early as 2005, it is expected that health care institutions in Nigeria, particularly the tertiary hospitals which represent the apex healthcare facilities, should be appropriately equipped to diagnose and manage these cases, fifteen (15) years after. Moreover, there is actually a dearth of data on the availability of these cardiac markers in tertiary care laboratories in Nigeria. Hence to provide this much needed data and contribute in filling the literature gap in this area, the authors therefore aimed at determining the extent of the central laboratory and point-of-care cardiac marker testing capacity of tertiary hospitals in Nigeria.

## Methods

### Study design

The study was a cross-sectional hospital-based comparative study carried out using a total population sampling of all government-owned operational, registered and licensed tertiary hospitals in Nigeria.

### Study Sites

The study was conducted between July 2019 and January 2020 and included government-owned tertiary hospitals located in the six (6) geopolitical zones of Nigeria; South-East, South-South, South-West, North-Central, North-East and North-West. These facilities were selected using the Nigeria Health Facility Registry developed by the Federal Ministry of Health. A search was conducted using the terms ‘operational’, ‘registered’, ‘licensed’, ‘tertiary’ and ‘public’. The search returned 47 entries. Nine (9) centers were excluded based on the exclusion criteria giving thirty-eight (38) centers as eligible for the study.

### Collection of data

Data were collected using a researcher-designed semi-structured self-administered questionnaire. The questionnaire was pretested using four privately-owned hospitals, which were eventually not part of the study. Feedback from the questionnaire pretesting was used to prepare the final draft which was approved by all authors before use. The first section of the questionnaire assessed socio-demographic characteristics of the respondent, location and bed-size of the institution while the second section assessed the cardiac marker testing services in the institution.

In each of the hospitals, a Pathologist, preferably a Chemical Pathologist, was sought out to fill the questionnaire. In hospitals without a Pathologist, either a resident doctor, a medical officer or a medical laboratory scientist but knowledgeable with MI and laboratory instrumentation, filled the questionnaire.

### Inclusion criteria

Government-owned operational, registered and licensed tertiary hospitals in Nigeria

### Exclusion criteria

- Government-owned non-tertiary, non-operational, unregistered and/or unlicensed hospitals

- Privately-owned hospitals

- Psychiatric hospitals

- Federal School of Dental Technology and Therapy

- National Blood Transfusion Service Centres

- Institute of Child Health Nutrition Units

- National Ear Care Center

- Annexed hospitals.

### Ethical considerations

Ethical clearance was obtained from Health Research Ethics Committee University of Nigeria Teaching Hospital, after review and approval of study proposal. Written informed consent was obtained from respondents after the purpose of the study was explained to them.

### Analysis of data

Data were entered into Microsoft Excel sheet and double-checked for accuracy. Statistical analysis was then carried out using Stata version 13 (Stata Corp., USA).

Descriptive analyses were presented in frequency tables as number and percentages while Continuous variables were presented as mean, standard deviation (SD), number and percentages. Fisher's exact test was used to determine the relationship of location and category of testing facilities with Turnaround Time (TAT). Statistical significance was presented using P-values and values < 0.05 were considered statistically significant.

## Results

### Soco-demographic characteristics of respondents

The Male: Female ratio of respondents was 3.3:1. None of the respondents had practiced in their various institutions for less than a year while the proportion of respondents who had practiced in their current institutions for greater than 10 years was 12 (35.3%). All respondents, apart from the medical officers, were from Pathology specialty with 27 (79.4%) being Consultants. The socio-demographic characteristics of respondents are as described in Table 1.

**Table 1:** Socio-demographic characteristics of respondents

Socio-demographic characteristics	Categories	Frequency (%)
**Sex**		
	Male	26 (76.5)
	Female	8 (23.5)
**Professional cadre**		
	Consultant	27 (79.4)
	Resident Doctor	4 (11.8)
	Medical Officer	2 (5.9)
	Medical Laboratory Scientist	1 (2.9)
**Length of Practice** **in current** **institution**		
	Less than 1 year	0 (0.0)
	1 – 5 years	12 (35.3)
	5 – 10 years	10 (29.4)
	>10 years	12 (35.3)

### Distribution and characteristics of surveyed hospitals

**Fig 1:**
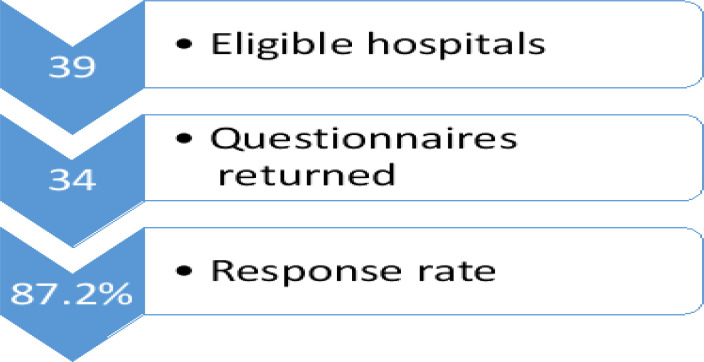
Flow Chart of Study Population

**Fig 2:**
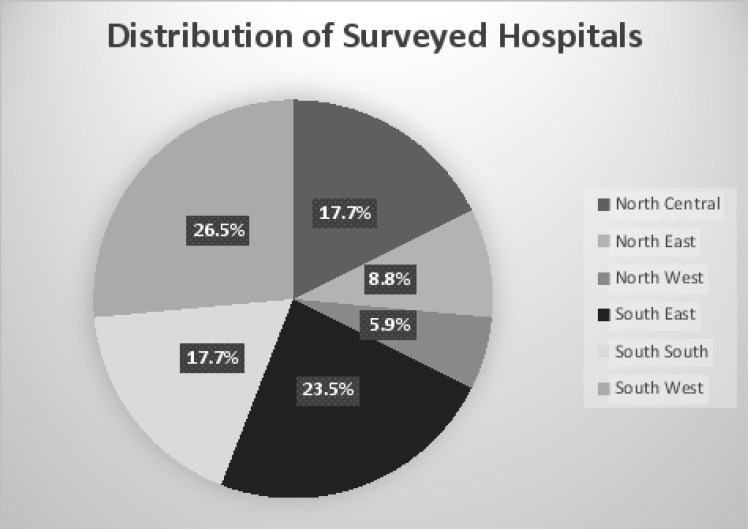
Distribution of surveyed hospitals among geopolitical zones in Nigeria

A total of thirty-four (34) tertiary hospitals participated in the study giving a response rate of 87.2% (Fig. 1). Figure 2 gives a description of the distribution of surveyed hospitals across the geopolitical zones of the country. The estimated bed size for most 12 (35.3%) of the surveyed institutions was greater than 500 (Table 2).

**Table 2:** Estimated bed size of institutions

S/N	Estimated bed size	Frequency (%)
1	Less than 100	1 (2.9)
2	100 – 200	8 (23.5)
3	200 – 300	5 (14.7)
4	300 – 400	3 (8.8)
5	400 – 500	5 (14.7)
6	Greater 500	12 (35.3)
7	Total	34 (100.0)

### Cardiac marker testing capacity and reasons for not testing

Of the surveyed hospitals, 19 (55.9%) hospitals were found to have a functional cardiac marker testing facility, either in the form of auto analyzer, semi-automated analyzer, POCT device or a combination of these, while 15 (44.1%) had none. For those without a facility, lack of funds to procure equipment, especially auto analyzer which is cost-intensive, was the reason given by majority 7 (20.6%). Other reasons given included equipment breakdown and lack of requests for tests. Concerning the location of the testing facility, of those with a functional facility, majority 12 (63.2%) indicated having it located in the central laboratory (Table 3). The number of functional facilities in each hospital varied with majority 8 (42.1%) having two (2) testing facilities. The number and the category of testing facilities are as depicted in Table 4. Different hospitals tested for different markers with Troponin I being the commonest, Table 5.

**Table 3:** Location of cardiac marker testing facility, N = 19

S/N	Location of testing facility (irrespective of category)	Frequency (%)
1	Central laboratory	12 (63.2)
2	Casualty/Emergency Unit	4 (21.1)
3	Cardiology clinic	1 (5.3)
4	Others like Research lab	5 (26.3)

Total > 100% due to some hospitals having more than one testing facility

**Table 4:** Number of testing facilities available in surveyed hospitals, N = 19

S/N	Number of testing facilities	Frequency (%)
1	One	7 (36.8)
2	Two	8 (42.1)
3	Three	3 (15.8)
4	Four	1 (5.3)
5	Five and above	0 (0.0)
	**Category of testing facility**	
1	Point-of-care only	5 (26.3%)
2	Auto analyzer only	5 (26.3%)
3	Point-of-care and Auto analyzer	5 (26.3%)
4	Semi-automated analyzer only	4 (21.1%)

**Table 5:** Different cardiac markers tested, N = 19

S/N	Cardiac markers tested	Frequency (%)
1	CKMB	13 (68.4)
2	Myoglobin	10 (52.6)
3	Troponin T	9 (47.4)
4	Troponin I	16 (84.2)
5	LDH	8 (42.1)

NB: Total > 100% due to some hospitals testing for more than one marker

### Turnaround time (TAT) and affordability of cardiac markers

**Fig 3:**
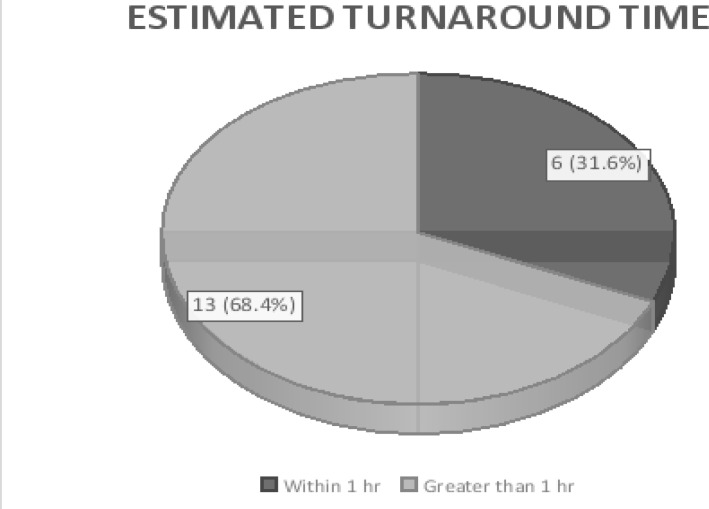
Estimated turn-around time for surveyed hospitals

Only 6 (31.58%) of participating hospitals had their turnaround time within 1 hour of test request, (Fig. 3). A statistically significant greater number of hospitals with testing facility located in emergency unit met the 1 hour timeline while the differences with category of testing device was not statistically significant, Table 6. Most respondents indicated N3000 – N4000 (7.87 – 10.50 USD) as the average cost of one cardiac marker, Table 7. Regarding this cost, a greater proportion of the respondents 13 (68.42%) felt the average cost of the test could not be afforded by many patients who required it; hence there was need for measures to reduce cost.

**Table 6:** Relationship of location and category of testing facilities with TAT, N = 22*

S/N		Turnaround time within 1 hour		P
	**Location of** **testing facility**	Yes Frequency (%)	No Frequency (%)	
**1**	Emergency Unit	4 (18.2)	0 (0.0)	0.00142
**2**	Central laboratory	2 (9.1)	10 (45.4)	
**3**	Others like research laboratory	0 (0.0)	6 (27.3)	
	**Category of** **testing facility**			
**1**	POCT	5 (22.7)	4 (18.2)	0.3054
**2**	Auto	4 (18.2)	6 (27.3)	
**3**	Semi autoanalyzer	0 (0.00)	3 (13.6)	

* Some hospitals have more than one testing facilities (multiple of a particular facility is counted as one unit in terms of location and category)

**Table 7:** Average cost of one cardiac marker, N = 19

S/N	Average cost in Naira (₦)	USD Equivalent^23^	Frequency (%)
1	1000 – 2000	2.62 – 5.25	1 (5.3)
2	2000 – 3000	5.25 – 7.87	3 (15.8)
3	3000 – 4000	7.87 – 10.50	7 (36.8)
4	4000 – 5000	10.50 – 13.12	2 (10.5)
5	Above 5000	Above 13.12	6 (31.6)
6	Total		19 (100.0)

## Discussion

The majority of the respondents in the present study were Consultants and resident doctors in Pathology specialty who are responsible for providing and interpreting the results of tests done in the laboratory, including cardiac marker tests. The use of this cadre of personnel was to ensure accurate information regarding the availability or otherwise of cardiac marker tests in participating hospitals. The skewed gender ratio in favour of males is not surprising as Pathology specialties are largely male-domi-nated ones, studies in America^24^ and also in Nigeria^25^ have documented this finding. The length of practice of respondents is important to ensure that respondents were not new in their work environment, hence had adequate information about their work place.

The present study noted that 19 (55.9%) of the surveyed hospitals had a functional cardiac marker testing device, either in the form of auto analyzer, semi-automated analyzer, POCT device or a combination of these. Though there is a dearth of data on this subject but a study done in northeastern Thailand^26^ documented that of the 18 hospitals surveyed, only one community hospital offered cTnI testing while cTnT and proBNP were found infrequently at province Regional Hospitals. It should however be noted that this Thailand study was conducted in rural hospitals and not in tertiary care hospitals which could explain the difference noted. The finding in the present study also contrasts with that of a study done in China^27^ which reported biomarker testing capability of 57.4% in 2001 and 96.3% in 2011. The Chinese study however included both secondary and tertiary care hospitals.

The availability of functional testing devices in the present study is adjudged to be low and grossly inadequate for the estimated Nigerian population of about 200 million,^28^ and for hospitals with estimated bed size mostly ranging from 100 to greater than 500. Timely and accurate diagnosis of ACS is essential for therapy and prognosis; hence this poor availability can indeed lead to missed diagnosis of ACS with its dire and oftentimes fatal consequences. In a survey^22^ conducted among ten (10) hospitals in the United States of America, it was documented that among the 889 patients with acute myocardial infarction, 19 (2.1%) were mistakenly discharged from the emergency department. Current study was among tertiary care hospitals which represent the apex healthcare institutions in the country and should handle referrals for such cases. Again this scenario of inadequate availability of testing facilities, especially central laboratory testing, is found fifteen years after observations of increasing prevalence of myocardial infarction were documented in Nigeria. Some respondents indicated lack of demand for tests as reason for unavailability of tests. This is a narrative which can be changed with improved communication between the laboratories and the clinicians, as sometimes the clinician is unaware of the current laboratory test repertoire. Moreover this communication can also be in the form of clinicians alerting the laboratories of the need to introduce these category of tests to assist the clinicians in providing optimal patient management.

Majority 12 (63.2%) of surveyed hospitals had their testing facilities located in the central laboratory. Only 4 (21.1%) facilities were located in the emergency unit. It was equally noted that only 6 (31.6%) hospitals had TAT of within 1hour while majority 13 (68.4%) had theirs longer than 1 hour. This falls short of the recommendation of the National Academy of Clinical Biochemistry Laboratory Medicine Practice Guidelines, which stipulated that laboratories should perform cardiac marker testing with a TAT of 1 hour, optimally 30 minutes or less.^29^ When a result takes more than 24 hours to be released, this limits the speed at which diagnosis is made, and the response rate to AMI and may consequently lead to increased morbidity and mortality.

Further analyses suggests that TAT is more influenced by location of testing facilities rather than category/type of facility. Though some studies^30^,^31^ have largely advocated for POC testing devices in the emergency unit, in the present study, location of testing devices, either POCT or auto analyzer, in the emergency unit helped greatly in achieving the 1 hour timeline. However, wherever POCT devices are used, there ought to be intermittent comparison of results with central laboratory testing to ensure reliability and accuracy of results generated with the POCT devices. Because most patients with acute chest pain are likely to present first to the emergency unit, it is plausible for hospitals to seriously consider locating at least one testing facility in the emergency unit. This is largely in a bid for timely and accurate diagnosis of ACS.

It is equally disheartening that only 16 (84.2%) and 9 (47.4%) of testing hospitals tested for Troponin I and Troponin T respectively despite cardiac troponin being central to the current universal definition of MI and essential in the diagnosis, proper risk stratification and overall management of ACS.

Majority 7 (36.8%) of the respondents indicated N3000 – N4000 as the average cost per cardiac marker in their institution. This is in a country where 40.1% of the citizens live below its poverty line of less than N137, 430 per year [less than N381.75 (1.00 USD equivalent^23^) per day] according to the National Bureau of Statistics.^32^ Consequently, a greater proportion of the respondents 13 (68.42%) equally opined that the cost of the test could not be afforded by many patients who required it, hence there is need for measures to reduce cost, probably with the provision of a viable health insurance system.

## Strengths and Limitation of Study

The major strength of the present study is its multicenter nature with representation of all geopolitical zones of the country. It equally serves to bridge the much needed gap in literature regarding this topic. However, being a cross-sectional study, it has the limitation of inability to determine effect and causality.

## Conclusion and recommendation

The central laboratory and point-of-care cardiac marker testing capacity of tertiary hospitals in Nigeria was found to be barely adequate with nearly a half of surveyed hospitals not offering the services. Among those offering the services, the TAT, the cost and the array of cardiac markers tested were also below expectation. A lot more effort is needed in this area by all stakeholders, to increase the testing capacity of hospitals especially with a background of increasing prevalence of MI in the country.
